# Orbital Myeloid Sarcoma as the Initial Presentation of Acute Myeloid Leukemia With Maturation in a Pediatric Patient: A Case Report

**DOI:** 10.7759/cureus.85026

**Published:** 2025-05-29

**Authors:** Moath Altarawneh, Alaa Alqasem, Mousa Qatawneh, Ayman Alhwayan, Firas Alsmadi, Mohammad Alsaaida, Amani Alrousan, Hind Alqatamin, Haneen Alrawashdeh, Maher Khader

**Affiliations:** 1 Paediatric Haematology-Oncology, Royal Medical Services-Queen Rania Children’s Hospital, Amman, JOR; 2 Paediatric Radiology, Royal Medical Services-Queen Rania Children’s Hospital, Amman, JOR; 3 Paediatric Ophthalmology, Royal Medical Services-Queen Rania Children’s Hospital, Amman, JOR; 4 Pathology and Laboratory Medicine, Royal Medical Services- Princess Iman Center for Research and Laboratory, Amman, JOR; 5 Pathology and Laboratory Medicine, Royal Medical Services-Princess Iman Center for Research and Laboratory, Amman, JOR

**Keywords:** acute myeloid leukemia, cross-border healthcare, orbital myeloid sarcoma, pediatric oncology, rare hematological malignancies

## Abstract

Acute myeloid leukemia (AML) is primarily a disease of adulthood but can also occur in children. Myeloid sarcoma (MS) is an uncommon extramedullary manifestation of AML, and orbital involvement is considered particularly rare, especially in pediatric cases.

We report a case of a four-year-old girl from Gaza who developed progressively worsening bilateral orbital swelling and ptosis over four months. Initial assessments, including an eyelid biopsy performed in Gaza, suggested a possible diagnosis of lymphangioma or rhabdomyosarcoma. However, limited access to advanced diagnostic resources and delays in cross-border referral significantly impacted definitive diagnosis. When she was finally referred to Jordan, advanced imaging revealed extensive bilateral retro-orbital masses, severe proptosis, and orbital wall erosion. Histopathological analysis confirmed that MS and bone marrow studies identified AML with maturation (French-American-British classification subtype M2 (FAB-M2)) and the translocation of t (8;21). Despite undergoing three cycles of chemotherapy, she tragically passed away just four months after diagnosis due to complications including encephalopathy and severe infection. This case highlights the aggressive nature of orbital MS in pediatric AML and the critical role of timely access to advanced medical care. It also highlights the devastating impact of healthcare inequities in conflict zones, where diagnostic and treatment delays can significantly worsen outcomes.

## Introduction

Myeloid sarcoma (MS), also known as granulocytic sarcoma or chloroma, is a rare tumor composed of immature myeloid cells that develop outside the bone marrow; it occurs in 2.5%-9.1% of acute myeloid leukemia (AML) cases and can affect various tissues, including bones, skin, lymph nodes, and the orbit [1]. In children, MS is even more uncommon, particularly as an initial presentation of AML, also most often associated with specific AML subtypes, including M2 (myeloblastic with maturation), M4 (myelomonocytic), and M5 (monoblastic/monocytic) [2].

Among head and neck sites, the orbit is the most frequent location for MS. However, it remains exceedingly rare, affecting less than 2% of pediatric AML cases, and typically presents as progressive proptosis accompanied by periorbital swelling or redness, which may be unilateral or bilateral [3]. In some cases, orbital MS appears before any systemic signs of AML, making early diagnosis particularly difficult. When hematologic involvement is absent, orbital MS is often misdiagnosed as other conditions, such as rhabdomyosarcoma, lymphoma, or neuroblastoma [4].

AML in children is uncommon, representing about 15%-20% of all childhood leukemias, with an annual incidence of two to four cases per 100,000 children [5]. MS involving the orbit carries a poor prognosis if not promptly recognized and treated, as it often progresses to systemic AML. Systemic chemotherapy is the cornerstone of treatment, as untreated MS frequently transforms into AML within a year [6]. Early diagnosis is crucial because prompt treatment can significantly improve outcomes.

This case report describes the unusual case of orbital MS being the first sign of AML in a four-year-old girl from Gaza. The diagnosis and subsequent treatment were greatly hindered by her quest for medical attention in a war-torn area. In particular, her diagnosis was delayed due to a lack of access to advanced imaging, a dearth of specialized pediatric oncology practitioners, slow cross-border referrals, and trouble obtaining basic prescription drugs.

In any event, this was one of those cases where cross-border cooperation proved beneficial. The patient was sent to Jordan, where the advanced diagnostic equipment and the available specialists definitively diagnosed her and started her on systemic chemotherapy. Her referral to Jordan provided desperate hope, but reaching specialized care did not help. Unfortunately, the disease continued to progress despite treatment, and she ultimately succumbed to it and passed away four months after her diagnosis. This case highlights the inequality of access to healthcare in resource-poor and conflict-affected zones. Despite hosting a large population of refugees, Jordan continues to show dedication and resilience in catering to vulnerable medical patients.

Through this case report, we aim to highlight the diagnostic and therapeutic challenges of orbital MS in pediatric patients, particularly when it presents as the initial manifestation of AML. We also emphasize the critical role of international cooperation in bridging healthcare gaps in conflict zones. This case serves as a call to action for more equitable healthcare access by highlighting the intersection of medical and humanitarian challenges. It underscores the transformative potential of cross-border cooperation in saving lives.

## Case presentation

A previously healthy four-year-old girl from Gaza first developed left periorbital swelling in April 2024. Over the next four months, the swelling worsened and spread to her right orbit, gradually affecting her vision and eye movements. Due to limited healthcare resources, she could only be evaluated by an ophthalmologist in August 2024. An orbital computed tomography (CT) scan revealed bilateral extraconal orbital masses, more prominent in the left orbit.

In August 2024, an incisional biopsy of the left eyelid mass was performed in Gaza, but histopathological findings were inconclusive, suggesting lymphangioma or rhabdomyosarcoma. Meanwhile, her symptoms progressed, her proptosis became more severe, and her eye movements grew increasingly restricted.

By October 2024, she was referred to Jordan for further evaluation. Upon arrival, she was alert, conscious, and not in any apparent respiratory distress. Her vital signs were stable: a temperature of 36.8°C, a heart rate of 96 beats per minute, a respiratory rate of 20 breaths per minute, and oxygen saturation of 99% on room air.

The rest of the physical examination was unremarkable. Cardiovascular assessment revealed a normal heart rate and rhythm without murmurs or abnormal heart sounds. Lung auscultation showed clear and equal breath sounds bilaterally. Abdominal examination revealed no hepatosplenomegaly, tenderness, or masses. Neurological assessment demonstrated an alert and oriented child with no focal deficits. No signs of lymphadenopathy, cyanosis, edema, or other abnormalities were noted in the peripheral examination.

However, her eye examination told a different story. She had significant bilateral proptosis with severely restricted eye movements in all directions. The swelling was particularly pronounced on the left, with conjunctival redness and noticeable protrusion of the conjunctival sac. Despite the severity of her condition, her pupils reacted normally to light, and there were no immediate signs of vision loss.

**Figure 1:**
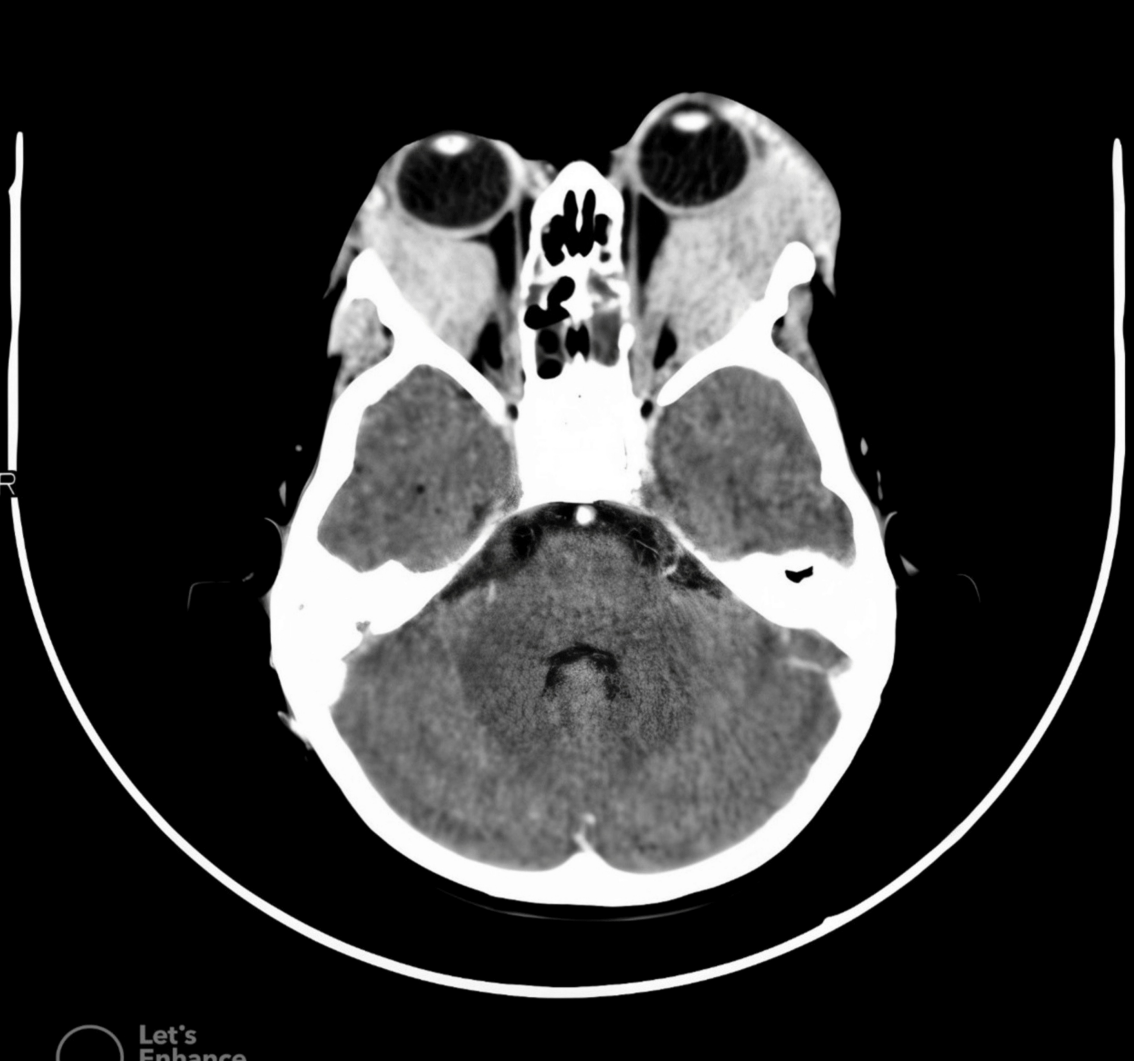
Axial CT Scan of the Orbits Showing Extensive Bilateral Retro-Orbital Masses; The Left Orbit Exhibits Severe Proptosis, and Orbital Wall Erosion Extends Inferiorly and Temporally, Demonstrating the Involvement of the Lacrimal Gland and Extraocular Muscles.

Advanced imaging in Jordan revealed extensive bilateral retro-orbital masses involving intra- and extraconal spaces. The tumor had caused severe proptosis and even orbital wall erosion. The masses extended inferiorly and temporally, inseparable from the lacrimal glands and extraocular muscles (Figure 1). Post-contrast CT imaging showed homogeneous enhancement. Imaging the brain, chest, and abdomen revealed no evidence of disease elsewhere.

Upon admission, routine laboratory tests, including complete blood count (CBC), kidney function tests (KFT), liver function tests (LFT), coagulation profile, and C-reactive protein (CRP), were largely within normal limits (Table 1). However, her erythrocyte sedimentation rate (ESR) was markedly elevated at 130 mm/hour. Of note, her hemoglobin was slightly reduced, consistent with mild anemia, and lactate dehydrogenase (LDH) was elevated, which may reflect increased cellular turnover.

**Table 1:** The Patient's Laboratory Test Results at Admission

Test	Result	Reference Range (4-Year-Old Child)
Complete Blood Count (CBC)		
White Blood Cells (WBC, ×10⁹/L)	16.9	5.0 – 14.5
Hemoglobin (Hb, g/dL)	9.4	11.5 – 13.5
Packed Cell Volume (PCV, %)	27.8	34 – 40
Mean Corpuscular Volume (MCV, fL)	85	75 – 87
Platelets (PLT, ×10⁹/L)	189	150 – 450
Absolute Neutrophil Count (ANC, ×10⁹/L)	4.8	1.5 – 8.5
Absolute Lymphocyte Count (ALC, ×10⁹/L)	6.2	2.0 – 9.0
Monocyte Count (×10⁹/L)	5.8	0.2 – 1.0
Kidney Function Tests (KFT)		
Creatinine (mg/dL)	0.35	0.3 – 0.7
Blood Urea Nitrogen (BUN, mg/dL)	8	5 – 18
Phosphorus (mg/dL)	4.4	3.8 – 6.5
Uric Acid (mg/dL)	6.1	2.0 – 5.5
Potassium (mmol/L)	4.1	3.5 – 5.0
Sodium (mmol/L)	139	135 – 145
Calcium (mg/dL)	10.5	8.5 – 10.8
Liver Function Tests (LFT)		
Aspartate Aminotransferase (AST, U/L)	19.3	10 – 40
Alanine Aminotransferase (ALT, U/L)	6.5	10 – 35
Total Bilirubin (mg/dL)	0.25	<1.0
Coagulation Profile		
Prothrombin Time (PT, sec)	13.4	11 – 15
Partial Thromboplastin Time (PTT, sec)	23.2	25 – 35
International Normalized Ratio (INR)	0.95	0.8 – 1.2
Other Tests		
Erythrocyte Sedimentation Rate (ESR, mm/hr)	130	<20
Lactate Dehydrogenase (LDH, U/L)	585	110 – 295
C-Reactive Protein (CRP, mg/dL)	2.6	<1.0
Beta-Human Chorionic Gonadotropin (β-HCG, IU/L)	0.1	<5.0
Alpha-Fetoprotein (AFP, ng/mL)	1.04	<10
Thyroid Stimulating Hormone (TSH, mIU/L)	1.5	0.5 – 4.5
Free Thyroxine (T4, ng/dL)	1.1	0.8 – 2.0

**Figure 2:**
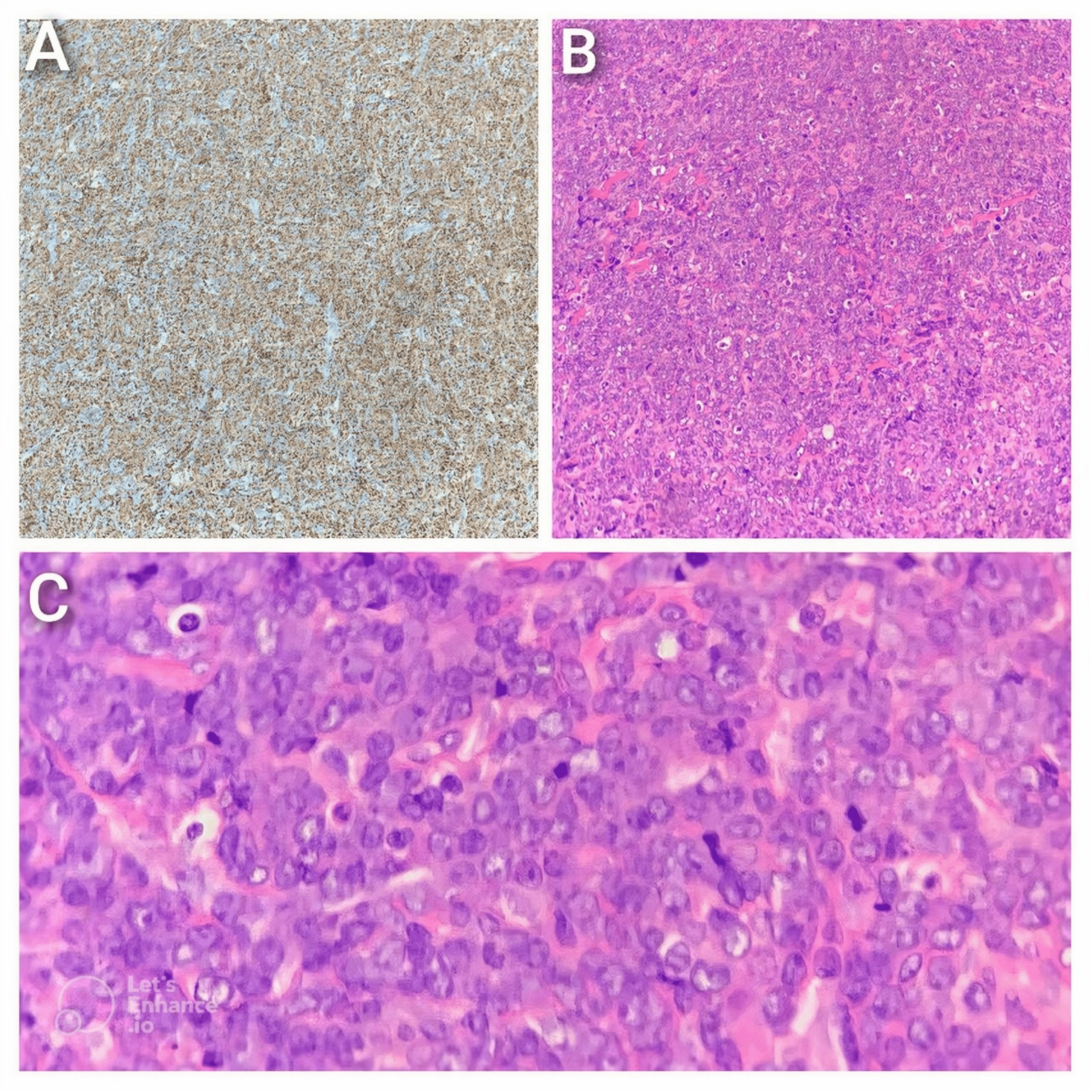
A. Immunohistochemical Staining Showing Strong Myeloperoxidase (MPO) Positivity, Confirming Myeloid Lineage of Immature Neoplastic Cells. B. H&E Stain at Intermediate Magnification Demonstrating Sheets of Neoplastic Cells Forming Cohesive Nests With a High Nuclear-to-Cytoplasmic (N/C) Ratio and Dispersed Chromatin. C. The High-Power View Shows Immature Blasts With Fine Nuclear Chromatin, Frequent Mitotic Figures, and Apoptotic Bodies Consistent With Aggressive Tumor Biology.

A repeat biopsy of the eyelid in Jordan provided the definitive answer: MS. Immunohistochemical staining confirmed the diagnosis, with strong positivity for myeloperoxidase (MPO), CD34, CD117, terminal deoxynucleotidyl transferase (TdT), B-cell lymphoma 2 (BCL2), and CD43 (Figure 2). The cells were negative for CD99, CD3, CD10, CD20, and CD22, effectively excluding lymphoid and other small round blue cell tumors from the differential diagnosis. Bone marrow aspiration and flow cytometry further established a diagnosis of AML with maturation (FAB-M2) and the t (8;21) translocation, with blast cells making up 30%-40% of the bone marrow.

**Figure 3:**
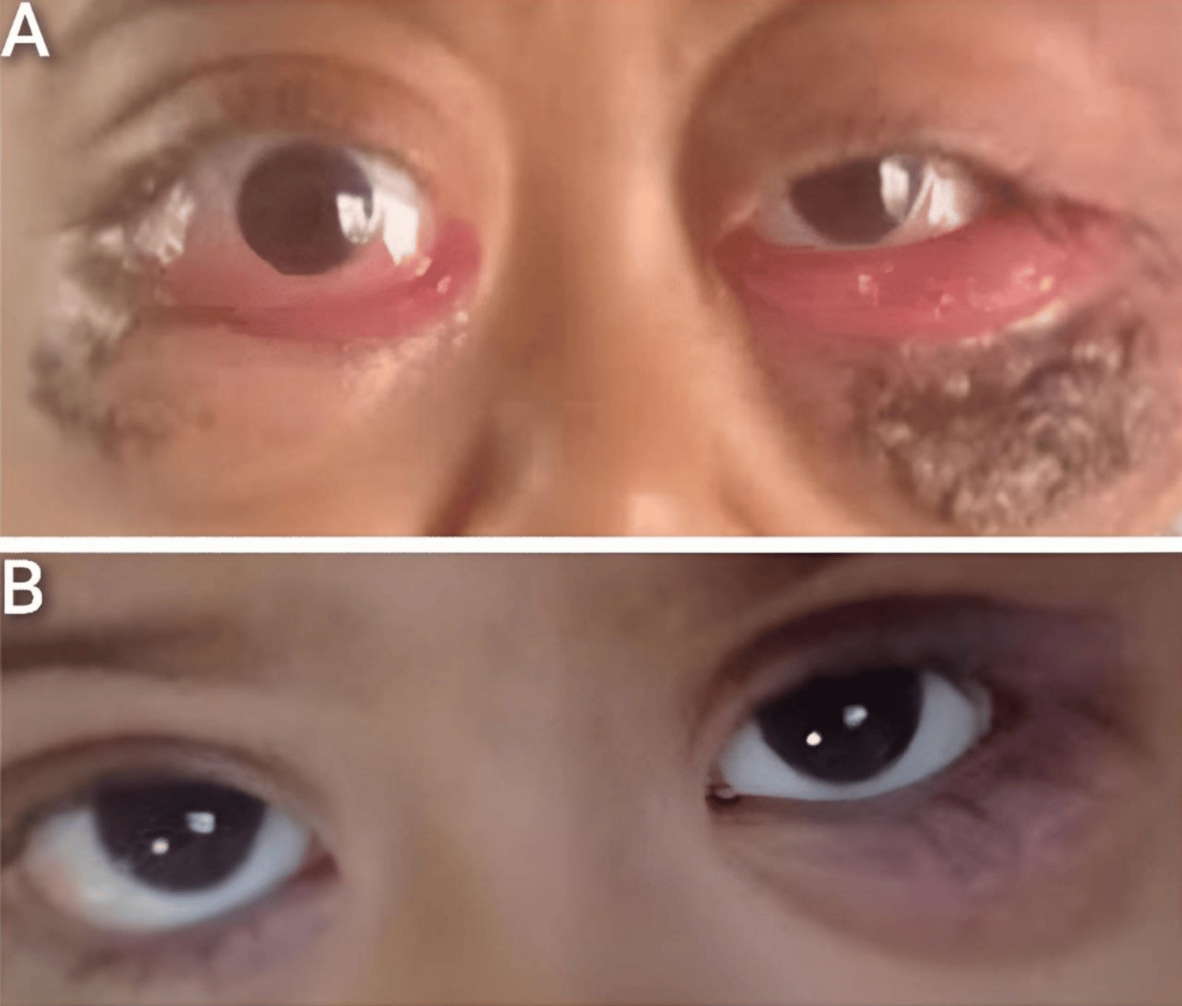
A. At Presentation: Left Eye Proptosis, Conjunctival Chemosis, and Conjunctival Sac Protrusion Are Evident. B. Post-first Cycle of Chemotherapy: Significant Reduction in Orbital Swelling and Proptosis, With Marked Clinical Improvement in Both Eyes.

After the definitive diagnosis was established, she was started on systemic chemotherapy. The first cycle consisted of low-dose cytarabine (200 mg/m²) daily for 10 days and doxorubicin (25 mg/m²), a total of three doses every other day, which significantly improved the orbital masses (Figure 3). After the first cycle, bone marrow examination showed 8%-10% blasts, and the t(8;21) translocation was negative, indicating the disease was not in remission.

The second cycle included low-dose cytarabine (200 mg/m²) for 10 doses daily, doxorubicin (25 mg/m²) for three doses every other day, and etoposide (100 mg/m²) for five days daily. Still, this cycle was complicated by prolonged myelosuppression, prolonged fever, and internal jugular vein thrombosis. Bone marrow examination following the second cycle showed 5%-8% blasts, with persistent disease and no remission. The third cycle consisted of high-dose cytarabine (3 gm/m²), five total doses daily, and mitoxantrone (10 mg/m²) on days one and five. Following this, the patient developed severe complications, including convulsions, altered mental status, and severe chest infection; she was admitted to the pediatric intensive care unit (PICU), where she deteriorated rapidly due to encephalopathy of unclear etiology (possibly leukemic infiltration, metabolic derangement, infection-related, or high-dose cytarabine) and passed away after a short time.

Her story is not just a medical case; it is a call to action. It highlights the urgent need for international advocacy in pediatric oncology to improve access to timely diagnosis, specialized care, and cross-border treatment pathways for children in conflict zones.

## Discussion

Orbital MS is a rare and aggressive manifestation of AML, occurring in less than 2% of pediatric AML cases [7]. With an estimated incidence of just 0.02 to 0.1 cases per million children annually [8], it is an uncommon diagnosis that often presents with symptoms mimicking more frequent orbital conditions such as lymphoma, rhabdomyosarcoma, aggressive orbital inflammatory syndrome, or IgG4-related orbital disease. Similarly, a study by Shields et al. [9] reported delays in diagnosis due to nonspecific symptoms like proptosis and periorbital swelling, which resemble more common orbital pathologies.

Initially, the lesion was thought to represent a rhabdomyosarcoma or lymphangioma based on biopsy results and imaging. These conditions are far more common in the pediatric population and can present similarly, especially when systemic signs are absent. This overlap often leads to diagnostic delay in rare cases like orbital MS. It's a reminder that when a child presents with persistent, progressive orbital swelling, particularly when bilateral, clinicians should consider MS in the differential diagnosis, even in the absence of hematologic findings. A high index of suspicion and early referral for immunohistochemistry and systemic workup can be pivotal.

MS arises when immature myeloid cells proliferate and infiltrate extramedullary tissues like the orbit. In this case, the initial manifestations were bilateral proptosis, periorbital swelling, and restricted eye movement. While unilateral orbital involvement is more common, as noted in a study of 25 cases by Daher et al. [10], our patient's rare bilateral involvement highlights the wide spectrum of clinical presentations in MS.

Proper imaging and histopathology are required to diagnose orbital MS confidently. In our case, the orbital CT scans demonstrated significant bilateral retro-orbital masses, which were in agreement with Zorn et al. [11], who claimed that the extraconal tumor growth had orbital bone destruction as a chief characteristic. Unfortunately, his conflict-afflicted part of the world did not receive timely specialized imaging and pathology services, which slowed the early diagnosis. In our case, immunohistochemical marker analysis of the tumor with the antibodies MPO, CD34, and CD117 proves the myeloid origin, parallel to Qian et al. [4], who noted that the positivity of the marker MPO was unparalleled.

The t(8;21) (q 22; q22) translocation, observed in our patient, is a hallmark of AML with maturation (FAB-M2), which occurs in 12%-15% of pediatric AML cases [12]. Johnston et al. [7] have previously reported that children with t(8;21) AML tend to respond well to chemotherapy, often achieving remission with standard treatment. However, our case did not show additional KIT mutations, which are associated with a higher relapse rate and poorer outcomes, as noted in Yang et al. [12].

The cornerstone of treatment for orbital MS is systemic chemotherapy, following standard protocols for AML. Most children with MS are put on induction therapy, which is a combination of cytarabine and an anthracycline, usually daunorubicin or idarubicin, to achieve remission. Our patient responded to the chemotherapy with anthracyclines, which supports the statements made by Johnston et al. [7] about the high rate of remission observed in pediatric MS cases treated with chemotherapy alone. However, despite a strong initial response, AML remains a high-risk disease, with relapse occurring in about 30%-40% of pediatric patients. While KIT mutation status informed our risk stratification, we were also guided by previous studies suggesting that molecular remission does not always correlate with clinical remission. Incorporating broader molecular profiling might offer better insight into treatment resistance and long-term prognosis in future cases.

While the t(8;21) translocation appeared to clear after the first chemotherapy cycle, remission was not achieved, as blast counts remained above 8%. One possible explanation could be the persistence of a subclone not carrying the translocation or simply that the disease was more chemoresistant than initially expected. Although KIT mutations were absent, other untested mutations may have played a role. We had planned to proceed with hematopoietic stem cell transplantation (HSCT) if remission was achieved after the third cycle, but unfortunately, the patient's condition deteriorated before this could be considered.

In the case of relapsed or unresponsive children, treatment is more aggressive. These cases are frequently treated with high doses of cytarabine, other targeted therapies, or HSCT. For patients with high-risk or relapsed AML, allogeneic hematopoietic stem cell transplantation (allo-HSCT) remains the most effective curative approach. This procedure not only replaces the diseased bone marrow with healthy hematopoietic cells but also induces a graft-versus-leukemia (GVL) effect, wherein donor immune cells recognize and eliminate residual leukemic cells, thereby reducing the risk of relapse. The GVL effect is a critical component of the therapeutic benefit of allo-HSCT in AML treatment [13]. However, the potency still relies on many conditions, such as identifying a compatible donor and reaching a state of minimum residual disease before the transplant.

Beyond chemotherapy and transplantation, new treatment options are emerging that could significantly improve outcomes for children with AML. Targeted therapies, such as FLT3 inhibitors, IDH1/2 inhibitors, and Menin inhibitors, are increasingly integrated into treatment plans based on a patient's specific genetic profile [14]. Meanwhile, immunotherapy is making strides in AML treatment. Chimeric antigen receptor (CAR) T-cell therapy has already transformed outcomes in B-cell acute lymphoblastic leukemia (B-ALL) and is now being explored for AML. Unlike B-ALL, where CD19 is a clear target, AML presents unique challenges due to the lack of leukemia-specific surface markers [15]. However, ongoing research into targets like CD33 and CD123 offers hope for the future. Although CAR-T therapy was not part of our patient's treatment, it holds promise for children with relapsed or refractory AML.

As our understanding of AML deepens, advances in molecular diagnostics and targeted therapies are allowing for more personalized treatment approaches. Studying the genetic and molecular makeup of MS, especially in cases involving the t(8;21) translocation, may help refine risk assessment and treatment decisions. However, it is crucial to address disparities in access to care beyond medical advancements. No child should have a better or worse chance at survival based on where they live. Bridging these gaps through stronger international medical collaborations, increased funding for pediatric oncology in underserved areas, and improved pathways for specialized care will be just as vital as the scientific breakthroughs.

This case illustrates the complex medical problem of diagnosing and managing a rare pediatric cancer. It simultaneously brings to light an issue regarding the inequity in the provision of healthcare services. In some parts of a conflict zone, children often present with advanced disease because of delays in diagnosis and barriers to accessing specialized care. Shamieh et al. [16] described how patients in conflict zones must deal with difficult logistical, economic, and institutional barriers. Sadly, this was the situation in our case in the conflict zone.

No child should have a better or worse chance at survival based on where they live. Bridging these gaps through stronger international medical collaborations, increased funding for pediatric oncology in underserved areas, and improved pathways for specialized care will be just as vital as the scientific breakthroughs. This aligns with the global call to integrate cancer services into humanitarian health responses in conflict and crisis settings, as emphasized by Casolino et al. [17].

## Conclusions

This case brings to light the complex medical, diagnostic, and humanitarian challenges faced when treating pediatric AML with orbital myeloid sarcoma, particularly in conflict-affected regions. The delayed diagnosis and limited access to advanced imaging and care. Early identification of orbital MS can significantly alter prognosis, especially when it precedes systemic signs of leukemia. Clinicians should maintain a high index of suspicion for AML in children presenting with bilateral proptosis or unexplained orbital masses. This report also emphasizes the life-saving potential of cross-border medical cooperation and the urgent need to bridge healthcare inequities. Children in underserved or conflict-affected areas deserve timely, specialized care. The outcome here might have been different if diagnosis and treatment had been initiated earlier. More than anything, this case is a call to action for clinicians, policymakers, and global health systems to ensure no child is left behind due to where they live.

## References

[1] Byrd JC, Edenfield WJ, Shields DJ, Dawson NA (1995). Extramedullary myeloid cell tumors in acute nonlymphocytic leukemia: a clinical review. J Clin Oncol.

[2] Pileri SA, Ascani S, Cox MC (2007). Myeloid sarcoma: clinico-pathologic, phenotypic and cytogenetic analysis of 92 adult patients. Leukemia.

[3] Kincaid MC, Green WR (1983). Ocular and orbital involvement in leukemia. Surv Ophthalmol.

[4] Qian X, Gigantelli JW, Abromowitch M, Morgan LA, Suh DW (2016). Myeloid sarcoma in the orbit. J Pediatr Ophthalmol Strabismus.

[5] De Kouchkovsky I, Abdul-Hay M (2016). Acute myeloid leukemia: a comprehensive review and 2016 update. Blood Cancer J.

[6] Neiman RS, Barcos M, Berard C, Bonner H, Mann R, Rydell RE, Bennett JM (1981). Granulocytic sarcoma: a clinicopathologic study of 61 biopsied cases. Cancer.

[7] Johnston DL, Alonzo TA, Gerbing RB, Lange BJ, Woods WG (2012). Superior outcome of pediatric acute myeloid leukemia patients with orbital and CNS myeloid sarcoma: a report from the Children's Oncology Group. Pediatr Blood Cancer.

[8] Samborska M, Derwich K, Skalska-Sadowska J, Kurzawa P, Wachowiak J (2016). Myeloid sarcoma in children - diagnostic and therapeutic difficulties. Contemp Oncol (Pozn).

[9] Shields JA, Stopyra GA, Marr BP, Shields CL, Pan W, Eagle RC Jr, Bernstein J (2003). Bilateral orbital myeloid sarcoma as initial sign of acute myeloid leukemia: case report and review of the literature. Arch Ophthalmol.

[10] Daher N, Duarte TF, Vetorasso GH, Capalbo RV, Bachiega TM (2018). Orbital myeloid sarcoma: a case report (Article in Portuguese). eOftalmo.

[11] Zorn KE, Cunningham AM, Meyer AE, Carlson KS, Rao S (2023). Pediatric myeloid sarcoma: more than just a chloroma—a review of clinical presentations, significance, and biology. Cancers (Basel).

[12] Yang J, Zhu X, Zhang H (2024). Prognostic factors of pediatric acute myeloid leukemia patients with t(8;21) (q22;q22): a single-center retrospective study. Children (Basel).

[13] Biederstädt A, Rezvani K (2023). How I treat high-risk acute myeloid leukemia using preemptive adoptive cellular immunotherapy. Blood.

[14] Lonetti A, Pession A, Masetti R (2019). Targeted therapies for pediatric AML: gaps and perspective. Front Pediatr.

[15] Turkalj S, Radtke FA, Vyas P (2023). An overview of targeted therapies in acute myeloid leukemia. Hemasphere.

[16] Shamieh O, Kutluk T, Fouad FM, Sullivan R, Mansour A (2023). Editorial: cancer care in areas of conflict. Front Oncol.

[17] Casolino R, Sullivan R, Jobanputra K (2025). Integrating cancer into crisis: a global vision for action from WHO and partners. Lancet Oncol.

